# The 1986 CODATA Recommended Values Of the Fundamental Physical Constants

**DOI:** 10.6028/jres.092.010

**Published:** 1987-04-01

**Authors:** E. Richard Cohen, Barry N. Taylor

**Affiliations:** Rockwell International, Science Center, Thousand Oaks, CA 91360; National Bureau of Standards, Gaithersburg, MD 20899

**Keywords:** CODATA, conversion factors, fundamental physical constants, least-squares adjustments, recommended values, Task Group on Fundamental Constants

## Abstract

This paper gives the values of the basic constants and conversion factors of physics and chemistry resulting from the 1986 least-squares adjustment of the fundamental physical constants as recently published by the CODATA Task Group on Fundamental Constants and as recommended for international use by CODATA. The new, 1986 CODATA set of recommended values replaces its predecessor published by the Task Group and recommended for international use by CODATA in 1973.

CODATA (Committee on Data for Science and Technology^1^) has recently published a report of the CODATA Task Group on Fundamental Constants prepared by the authors [1]^2^ under the auspices and guidance of the Task Group. The report summarizes the 1986 least-squares adjustment of the fundamental physical constants and gives a set of self-consistent values for the basic constants and conversion factors of physics and chemistry derived from that adjustment. Recommended for international use by CODATA, this 1986 set of values is reprinted here for the convenience of the many readers of the *Journal of Research of the National Bureau of Standards* and to assist in its dissemination throughout the scientific and technological communities. The 1986 CODATA set entirely replaces its immediate predecessor, that recommended for international use by CODATA in 1973. This set was based on the 1973 least-squares adjustment of the fundamental physical constants which was also carried out by the authors under the auspices and guidance of the Task Group [2,3].

As in previous least-squares adjustments of the constants [3,4,5], the data for the 1986 adjustment were divided into two groups: auxiliary constants and stochastic input data. Examples of the 1986 auxiliary constants are the speed of light in vacuum *c*≡299792458 m/s; the permittivity of vacuum *μ*_0_≡4*π*×10^−7^ N/A^2^; the Rydberg constant for infinite mass *R*_∞_; and the quantity *E*≡483594.0×10^9^ Hz/V which is equal numerically to the value of the Josephson frequency-voltage ratio 2 *e/h* (*e* is the elementary charge and *h* is the Planck constant) adopted in 1972 by the Consultative Committee on Electricity of the International Committee of Weights and Measures for defining laboratory representations of the volt [6,7]. Quantities in this category are either defined constants such as *c*, *μ*_0_, and *E* with no uncertainty, or constants such as *R*_∞_ with assigned uncertainties sufficiently small in comparison with the uncertainties assigned the stochastic input data with which they are associated in the adjustment that they can be taken as exact (i.e., their values are not subject to adjustment in contrast to the stochastic data). In the 1986 adjustment the uncertainty of each auxiliary constant was no greater than 0.02 parts-per-million or ppm.^3^ In contrast, the uncertainties assigned the 38 items of stochastic input data considered in the 1986 adjustment were in the range 0.065 to 9.7 ppm. (The 38 items were of 12 distinct types with the number of items of each type ranging from one to six.) Examples of such data are measurements of the proton gyromagnetic ratio $\gamma_{p}^{\prime }$ (uncertainty in the range 0.24 to 5.4 ppm), the molar volume of silicon *M*(Si)/*ρ*(Si) (1.15 ppm), and the quantized Hall resistance *R*_H_*=h/e*^2^ (0.12 to 0.22 ppm).

Because new results which can influence a least- squares adjustment of the constants are reported continually, it is always difficult to choose an optimal time at which to carry out a new adjustment and to revise the recommended values of the constants. In the present case, all data available up to 1 January 1986 were considered for inclusion, with the recognition that any additional changes to the 1973 recommended values that might result by taking into account more recent data would be much less than the changes resulting from the data available prior to that date.

Each of the 38 items of stochastic data are expressed (using the auxiliary constants as necessary) in terms of five quantities that serve as the “unknowns” or variables of the 1986 adjustment. These are *α*^−1^, the inverse fine-structure constant; *K*_v_, a dimensionless quantity relating the SI (International System of Units) volt V to the unit of voltage V_76-BI_ maintained at the International Bureau of Weights and Measures (BIPM) using a value of the Josephson frequency-voltage ratio equal numerically to *E*: V_7__6-BI_=*K*_V_ V, and thus *2e/h=E/K*_v_; *K*_Ω_, a dimensionless quantity relating the SI ohm to the BIPM as-maintained unit of resistance as it existed on 1 January 1985, Ω_BI85_, based on the mean resistance of a particular group of wire-wound precision resistors: Ω_BI85_=*K*_Ω_ Ω; *d*_220_, the (220) lattice spacing of a perfect crystal of pure silicon at 22.5 °C in vacuum; and *μ_μ_*/*μ*_p_, the ratio of the magnetic moment of the muon to that of the proton. “Best” values in the least-squares sense for these five quantities, with their variances and covariances, are thus the immediate output of the adjustment.

After a thorough analysis using a number of least-squares algorithms, the initial group of 38 items of stochastic input data was reduced to 22 items by deleting those that were either highly inconsistent with the remaining data or had assigned uncertainties so large that they carried negligible weight. The adjusted values of the five unknowns, and hence all the other 1986 recommended values that were subsequently derived from them (with the aid of the auxiliary constants), are therefore based on a least-squares adjustment with 17 degrees of freedom.

The 1986 adjustment represents a major advance over its 1973 counterpart; the uncertainties of the recommended values have been reduced by roughly an order of magnitude due to the enormous advances made throughout the precision measurement-fundamental constants field in the last dozen years. This can be seen from the following comparison of the 1973 and 1986 recommended values for the inverse fine-structure constant *α*^−1^, the elementary charge *e*, the Planck constant *h*, the electron mass *m*_e_, the Avogadro constant *N*_A_, the proton electron mass ratio *m*_p_/*m*_e_, the Faraday constant *F*, and the Josephson frequency-voltage ratio 2 *e*/*h*:

**Table:** 

Quantity	Uncertainty of recommended value in ppm		Change in 1973 recommended value in ppm resulting from 1986 adjustment
	1973	1986	
*α*^−1^	0.82	0.045	−0.37
*e*	2.9	0.30	−7.4
*h*	5.4	0.60	−15.2
*m*_e_	5.1	0.59	−15.8
*N*_A_	5.1	0.59	+15.2
*m*_p_*/m*_e_	0.38	0.020	+0.64
*F*	2.8	0.30	+7.8
2*e*/*h*	2.6	0.30	+7.8

It is also clear from this comparison that unexpectedly large changes have occurred in the 1973 recommended values of a number of these constants (i.e., a change which is large relative to the uncertainty assigned the 1973 value). These changes are a direct consequence of the 7.8 ppm decrease from 1973 to 1986 in the quantity *K*_v_ and the high correlation between *K*_v_ and the calculated values of *e*, *h, m*_e_, *N*_A_, and *F.* Since 2*e/h=E/K*_v_, the 1986 value of *K*_v_ also implies that the value of the Josephson frequency-voltage ratio adopted by the Consultative Committee on Electricity in 1972, which was believed to be consistent with the SI value and which most national standards laboratories adopted to define and maintain their laboratory unit of voltage, is actually 7.8 ppm smaller than the SI value. This unsatisfactory situation should be rectified in the near future [8,9].

The large change in *K*_v_ and hence in many other quantities between 1973 and 1986 would have been avoided if two determinations of *F* which seemed to be discrepant with the remaining data had not been deleted in the 1973 adjustment. In retrospect, the disagreement was comparatively mild. In view of this experience it is important to recognize that there are no similar disagreements in the 1986 adjustment; the measurements which were deleted were so discrepant that they obviously could not be correct, or of such low weight that if retained the adjusted values of the five unknowns would change negligibly. Thus, it is unlikely that any alternate evaluation of the data considered in the 1986 least-squares adjustment could lead to significant changes in the 1986 recommended values. Moreover, the quality of the 1986 data and its redundancy would seem to preclude future changes in the 1986 recommended values relative to their uncertainties comparable to the changes which occurred in the 1973 values.

The 1986 recommended values of the fundamental physical constants are given in five tables. Table 1 is an abbreviated list containing the quantities which should be of greatest interest to most users. Table 2 is a much more complete compilation. Table 3 is a list of related “maintained units and standard values,” while table 4 contains a number of scientifically, technologically, and metrologically useful energy conversion factors. Finally, table 5 is an extended covariance matrix containing the variances, covariances, and correlation coefficients of the unknowns and a number of different constants (included for convenience) from which the like quantities for other constants may be readily calculated.^4^ Such a matrix is necessary, of course, because the variables in a least-squares adjustment are statistically correlated. Thus, with the exception of quantities which depend only on auxiliary constants, the uncertainty associated with a quantity calculated from other constants in general can be found only with the use of the full covariance matrix.

To use table 5, note that the covariance between two quantities *Q_k_* and *Q_s_* which are functions of a common set of variables *x_i_*(*i* = 1,…, *N*) is given by

$$v_{ks}=\sum_{i,j=1}^{N}\frac{\partial Q_{k}}{\partial x_{i}}\frac{\partial Q_{s}}{\partial x_{j}}v_{ij}$$ (1)

where *v_ij_* is the covariance of *x_i_*·and *x_j_.* In this general form, the units of *v_ij_* are the product of the units of *x_i_* and *x_j_* and the units of *v_ks_* are the product of the units of *Q_k_* and *Q_s_.* For most cases of interest involving the fundamental constants, the variables x*_i_* may be taken to be the fractional change in the physical quantity from some fiducial value, and the quantities *Q* can be expressed as powers of physical constants Z*_j_* according to

$$Q_{k}=q\prod_{j=1}^{N}Z_{j}^{Y_{kj}},$$ (2)

where *q* is a numerical factor. If the variances and covariances are then expressed in relative units, eq (1) becomes

$$v_{ks}=\sum_{i,j=1}^{N}Y_{ki}Y_{sj}v_{ij},$$ (3)

where the *v_ij_* are to be expressed, for example, in (parts in 10^9^)^2^. Equation (3) is the basis for the expansion of the covariance matrix to include *e, h, m*_e_, *N*_A_, and *F.*

In terms of correlation coefficients defined by *r_ij_*≡*v_ij_*(*v_ii_v_jj_*)^−1/2^≡*v_ij_*/*ϵ_i_ϵ_j_*, where *ϵ_i_*, is the standard deviation $\left(\epsilon_{i}^{2}=v_{ii}\right)$, we may write, from eq (3),

$$\epsilon_{k}^{2}=\sum_{i=1}^{N}Y_{ki}^{2}\epsilon_{i}^{2}+2\sum_{j<i}^{N}Y_{ki}Y_{kj}r_{ij}\epsilon_{i}\epsilon_{j},$$ (4)

where the standard deviations are to be expressed in relative units.

As an example of the use of table 5, consider the calculation of the uncertainty of the Bohr magneton μ_B_*=eħ*/2*m*_e_ (*ħ =h*/2*π*). In terms of the variables of the 1986 adjustment this ratio is given by

$$\mu_{B}={\left[2\pi \mu_{0}R_{\infty }E\right]}^{-1}{\left(\alpha^{-1}\right)}^{-3}K_{V},$$ (5)

where the quantities in brackets are auxiliary constants taken to be exact. Using eq (3) and letting *α*^−1^ correspond to *i* = 1 and *K*_v_ to *i* =2 gives^5^

$$\epsilon_{\mu_{B}}^{2}=Y_{1}^{2}v_{11}+2Y_{1}Y_{2}v_{12}+Y_{2}^{2}v_{22}.$$ (6)

Comparing eq (5) with eq (2) yields *Y*_l_ = −3 and *Y*_2_ = 1. Thus eq (6) and table 5 lead to

$$\epsilon_{\mu_{B}}^{2}=[9(1997)-6(-1062)+1(87988)]\times {\left(10^{-9}\right)}^{2}$$ (7)

or $\epsilon_{\mu_{B}}=0.335\;\mathrm{ppm}$. An alternate approach is to evaluate *eħ*/2*m*_e_ directly from table 5; then *e* corresponds to *i* = 5, *h* to *i* = 6, and *m_e_* to *i* = 7 with *Y*_5_ = *Y*_6_ = 1and *Y*_7_= −1. Then

$$\begin{array}{l} \epsilon_{\mu_{B}}^{2}=Y_{5}^{2}v_{55}+2Y_{5}Y_{6}v_{56}+Y_{6}^{2}v_{66}\\ \;+2Y_{5}Y_{7}v_{57}+2Y_{6}Y_{7}v_{67}+Y_{7}^{2}v_{77} \end{array}$$ (8a)

$$\begin{array}{l} =[1(92109)+2(181159)+1(358197)\\ -2(175042)-2(349956)\\ +1(349702)]\times (10^{-9}{)}^{2} \end{array}$$ (8b)

which also yields $\epsilon_{\mu_{B}}=0.335\;\mathrm{ppm}$.

## Figures and Tables

**Table 1:** Summary of the 1986 recommended values of the fundamental physical constants An abbreviated list of the fundamental constants of physics and chemistry based on a least-squares adjustment with 17 degrees of freedom. The digits in parentheses are the one-standard-deviation uncertainty in the last digits of the given value. Since the uncertainties of many of these entries are correlated, the full covariance matrix must be used in evaluating the uncertainties of quantities computed from them.

Quantity	Symbol	Value	Units	Relative Uncertainty (ppm)
speed of light in vacuum	*c*	299792458	ms^−1^	(exact)
permeability of vacuum	*μ*_o_	4π × 10^−7^	N /A^−2^	
		=12.566370614…	10^−7^N A^−2^	(exact)
permittivity of vacuum	*ϵ*_o_	1/*μ*_o_*c*^2^		
		=8.854187817…	10^−12^ F m^−1^	(exact)
Newtonian constant of gravitation	*G*	6.67259(85)	10^−11^ m^3^ kg^−1^ s^−2^	128
Planck constant	*h*	6.6260755(40)	10^−34^ J s	0.60
*h*/2π	*ħ*	1.05457266(63)	10^−34^J s	0.60
elementary charge	*e*	1.602 17733(49)	10^−19^ C	0.30
magnetic flux quantum, *h*/2*e*	Φ_ο_	2.06783461(61)	10^−15^ Wb	0.30
electron mass	*m*_e_	9.1093897(54)	10^−31^ kg	0.59
proton mass	*m*_p_	1.672 6231(10)	10^−27^ kg	0.59
proton-electron mass ratio	*m*_p_/*m*_e_	1836.152701(37)		0.020
fine-structure constant, *μ*_o_*ce*^2^/2*h*	*α*	7.29735308(33)	10^−3^	0.045
inverse fine-structure constant	*α*^−1^	137.0359895(61)		0.045
Rydberg constant, *m*_e_*cα*^2^/2*h*	*R*_∞_	10973731.534(13)	m^−1^	0.0012
Avogadro constant	*N*_A_, *L*	6.0221367(36)	10^23^ mol^−1^	0.59
Faraday constant, *N*_A_*e*	*F*	96485.309(29)	C mol^−1^	0.30
molar gas constant	*R*	8.314510(70)	J mol^−1^ K^−1^	8.4
Boltzmann constant, *R*/*N*_N_	*k*	1.380658(12)	10^−23^J K^−1^	8.5
Stefan-Boltzmann constant, (π^2^/60)*k*^4^/*ħ*^3^*c*^2^	*σ*	5.67051(19)	10^−8^W m^−2^ K^−4^	34
Nοn-SI units used with SI				
electron volt, (*e*/C)J = {e}J	eV	1.602177 33(49)	10^−19^ J	0.30
(unified) atomic mass unit, $1\;u=m_{u}=\frac{1}{12}m{(}^{12}C)$	u	1.6605402(10)	10^−27^ kg	0.59

**Table 2:** The 1986 recommended values of the fundamental physical constants This list of the fundamental constants of physics and chemistry is based on a least-squares adjustment with 17 degrees of freedom. The digits in parentheses are the one-standard-deviation uncertainty in the last digits of the given value. Since the uncertainties of many of these entries are correlated, the full covariance matrix must be used in evaluating the uncertainties of quantities computed from them.

Quantity	Symbol	Value	Units	Relative Uncertainty (ppm)
GENERAL CONSTANTS				
Universal Constants				
speed of light in vacuum	*c*	299792458	ms^−1^	(exact)
permeability of vacuum	*μ*_o_	4π × 10^−7^ = 12.566370614…	N A^−2^ 10^−7^N A^−2^	(exact)
permittivity of vacuum	*ϵ*_o_	1/*μ*_o_*c*^2^ =8.854187817…	10^−12^ F m^−1^	(exact)
Newtonian constant of gravitation	*G*	6.67259(85)	10^−11^ m^3^ kg^−1^ s^−2^	128
Planck constant	*h*	6.6260755(40)	10^−34^ J s	0.60
in electron volts, *h*/{*e*}		4.1356692(12)	10^−15^ eVs	0.30
*h*/2π	*ħ*	1.05457266(63)	10^−34^ J s	0.60
in electron volts, *ħ*/{*e*}		6.5821220(20)	10^−16^ eV s	0.30
Planck mass, ${\left(\hbar c/G\right)}^{\frac{1}{2}}$	*m*_P_	2.17671(14)	10^−8^ kg	64
Planck length, $\hbar /m_{p}c={\left(\hbar G/c^{3}\right)}^{\frac{1}{2}}$	*l*_P_	1.61605(10)	10^−35^ m	64
Planck time, $l_{P}/c={\left(\hbar G/c^{5}\right)}^{\frac{1}{2}}$	*t*_P_	5.39056(34)	10^−44^ s	64
Electromagnetic Constants				
elementary charge	*e*	1.60217733(49)	10^−19^C	0.30
	*e*/*h*	2.41798836(72)	10^14^ A J^−1^	0.30
magnetic flux quantum, *h*/2*e*	Φ_ο_	2.06783461(61)	10^−15^ Wb	0.30
Josephson frequency-voltage ratio	2*e*/*h*	4.8359767(14)	10^14^ Hz V^−1^	0.30
quantized Hall conductance	*e*^2^/*h*	3.87404614(17)	10^−5^ S	0.045
quantized Hall resistance, *h*/*e*^2^ = *µ*_o_*c*/2*α*	*R*_H_	25812.8056(12)	Ω	0.045
Bohr magneton, e*ħ*/2*m*_e_	*μ*_B_	9.2740154(31)	10^−24^ JT^−1^	0.34
in electron volts, *μ*_B_/{*e*}		5.78838263(52)	10^−5^ eV T^−1^	0.089
in hertz, *μ*_B_*/h*		1.39962418(42)	10^10^ Hz T^−1^	0.30
in wavenumbers, *μ*_B_/*hc*		46.686437(14)	m^−1^ T^−^^1^	0.30
in kelvins, *μ*_B_/*k*		0.6717099(57)	K T^−1^	8.5
nuclear magneton, *eħ/*2*m*_p_	*μ*_N_	5.0507866(17)	10^−27^J T^−1^	0.34
in electron volts, *μ*_N_/{*e*}		3.15245166(28)	10^−8^ eVT^−1^	0.089
in hertz, *μ*_N_/*h*		7.6225914(23)	MHz T^−1^	0.30
in wavenumbers, *μ*_N_*/hc*		2.54262281(77)	10^−2^ m^−1^ T^−1^	0.30
in kelvins, *μ*_N_/*k*		3.658246(31)	10^−4^ K T^−1^	8.5
ATOMIC CONSTANTS				
fine-structure constant, *μ*_o_*ce*^2^/2*h*	*α*	7.29735308(33)	10^−3^	0.045
inverse fine-structure constant	*α*^−1^	137.0359895(61)		0.045
Rydberg constant, *m*_e_*cα*^2^/2*h*	*R*_∞_	10 973731.534(13)	m^−1^	0.0012
in hertz, *R*_∞_*c*		3.2898419499(39)	10^15^ Hz	0.0012
in joules, *R*_∞_*hc*		2.1798741(13)	10^−18^ J	0.60
in eV, *R*_∞_*hc*/{*e*}		13.6056981(40)	eV	0.30
Bohr radius, *α*/4*π R*_∞_	*a*_o_	0.529177249(24)	10^−10^ m	0.045
Hartree energy, *e*^2^/4*πϵ*_o_*α*_o_ = 2*R*_∞_*hc*	*E*_h_	4.3597482(26)	10^−18^ J	0.60
in eV, *E*_h_/{*e*}		27.2113961(81)	eV	0.30
quantum of circulation	*h*/2*m*_e_	3.63694807(33)	10^−4^ m^2^ s^−1^	0.089
	*h*/*m*_e_	7.27389614(65)	10^−4^ m^2^ s^−1^	0.089
Electron				
electron mass	*m*_e_	9.1093897(54)	10^−31^ kg	0.59
		5.48579903(13)	10^−4^ u	0.023
in electron volts, *m*_e_*c*^2^/{*e*}		0.51099906(15)	MeV	0.30
electron–muon mass ratio	*m*_e_/*m_μ_*	4.83633218(71)	10^−3^	0.15
electron–proton mass ratio	*m*_e_/*m*_p_	5.44617013(11)	10^−4^	0.020
electron–deuteron mass ratio	*m*_e_/*m*_d_	2.72443707(6)	10^−4^	0.020
electron–*α*-particle mass ratio	*m*_e_/*m_α_*	1.370 93354(3)	10^−4^	0.021
electron specific charge	*−e/m*_e_	*−*1.75881962(53)	10^11^ C kg^−1^	0.30
electron molar mass	*M*(e), *M*_e_	5.48579903(13)	10^−7^ kg/mol	0.023
Compton wavelength, *h/m*_e_*c*	*λ*_C_	2.42631058(22)	10^−12^ m	0.089
*λ*_C_/2*π* = *αa*_o_ = *α*^2^/4π *R*_∞_	*λ*_C_	3.86159323(35)	10^−13^ m	0.089
classical electron radius, *α*^2^*a*_o_	*r*_e_	2.81794092(38)	10^−15^ m	0.13
Thomson cross section, $\left(8\pi /3\right)r_{e}^{2}$	*σ*_e_	0.665 24616(18)	10^−28^ m^2^	0.27
electron magnetic moment	*μ*_e_	928.47701(31)	10^−26^ J T^−^^1^	0.34
in Bohr magnetons	*μ*_e_/*μ*_B_	1.001159652193(10)		l × 10^−5^
in nuclear magnetons	*μ*_e_/*μ*_N_	1838.282000(37)		0.020
electron magnetic moment				
anomaly, *μ*_e_/*μ*_B_ − 1	*a*_e_	1.159652193(10)	10^−3^	0.0086
electron g-factor, 2(1 + *a*_e_)	*g*_e_	2.002319304386(20)		1×10^−5^
electron-muon				
magnetic moment ratio	*μ*_e_*/μ_μ_*	206.766967(30)		0.15
electron–proton				
magnetic moment ratio	*μ*_e_/*μ*_P_	658.2106881(66)		0.010
Muon				
muon mass	*m_μ_*	1.8835327(11)	10^−28^ kg	0.61
		0.113428913(17)	u	0.15
in electron volts, *m_μ_c*^2^/{*e*}		105.658389(34)	MeV	0.32
muon–electron mass ratio	*m_μ_*/*m*_e_	206.768262(30)		0.15
muon molar mass	*M*(*μ*),*M_μ_*	1.13428913(17)	10^−4^ kg/mol	0.15
muon magnetic moment	*μ_μ_*	4.4904514(15)	10^−26^ J T^−1^	0.33
in Bohr magnetons,	*μ_μ_*/*μ*_B_	4.84197097(71)	10^−3^	0.15
in nuclear magnetons,	*μ_μ_*/*μ*_N_	8.8905981(13)		0.15
muon magnetic moment anomaly,				
[*μ_μ_*/(*eħ/2m_μ_*)] − 1	*a_μ_*	1.1659230(84)	10^−3^	7.2
muon g-factor, 2(1 *+ a_μ_*) muon–proton	*g_μ_*	2.002331846(17)		0.0084
magnetic moment ratio	*μ_μ_*/*μ*_p_	3.18334547(47)		0.15
Proton				
proton mass	*m*_p_	1.6726231(10)	10^−27^ kg	0.59
		1.007276470(12)	u	0.012
in electron volts, *m*_p_*c*^2^/{*e*}		938.27231(28)	MeV	0.30
proton–electron mass ratio	*m*_p_/*m*_e_	1836.152701(37)		0.020
proton–muon mass ratio	*m*_p_*/m_μ_*	8.8802444(13)		0.15
proton specific charge	*e/m*_p_	9.5788309(29)	10^7^ Ckg^−1^	0.30
proton molar mass	*M*(p), *M*_p_	1.007276470(12)	10^−3^ kg/mol	0.012
proton Compton wavelength, *h*/*m*_p_*c*	*λ*_C,p_	1.32141002(12)	10^−15^ m	0.089
*λ*_C,p_/2*π*	*λ*_C,p_	2.10308937(19)	10^−16^ m	0.089
proton magnetic moment	*μ*_p_	1.41060761(47)	10^−26^ JT^−1^	0.34
in Bohr magnetons	*μ*_P_/*μ*_B_	1.521032202(15)	10^−3^	0.010
in nuclear magnetons	*μ*_p_/*μ*_N_	2.792847386(63)		0.023
diamagnetic shielding correction				
for protons in pure water, spherical sample, 25 °C, $1-\mu_{p}^{\prime }/\mu_{p}$	*^σ^*H_2_O	25.689(15)	10^−6^	–
shielded proton moment	$\mu_{p}^{\prime }$	1.41057138(47)	10^−26^ J T^−1^	0.34
(H_2_O, sph., 25 °C)				
in Bohr magnetons	$\mu_{p}^{\prime }/\mu_{B}$	1.520993129(17)	10^−3^	0.011
in nuclear magnetons	$\mu_{p}^{\prime }/\mu_{N}$	2.792775642(64)		0.023
proton gyromagnetic ratio	*γ*_p_	26752.2128(81)	10^4^ s^−1^ T^−1^	0.30
	*γ*_p_/2*π*	42.577469(13)	MHz T^−1^	0.30
uncorrected (H_2_O, sph., 25 °C)	$\gamma_{p}^{\prime }$	26751.5255(81)	10^4^ s^−1^ T^−1^	0.30
	$\gamma_{p}^{\prime }/2\pi$	42.576375(13)	MHz T^−1^	0.30
Neutron				
neutron mass	*m*_n_	1.6749286(10)	10^−27^ kg	0.59
		1.008664904(14)	u	0.014
in electron volts, *m*_n_*c*^2^/{*e*}		939.56563(28)	Mev	0.30
neutron–electron mass ratio	*m*_n_/*m*_e_	1838.683662(40)		0.022
neutron–proton mass ratio	*m*_n_/*m*_p_	1.001378404(9)		0.009
neutron molar mass	*M*(n), *M*_n_	1.008664904(14)	10^−3^ kg/mol	0.014
neutron Compton wavelength, *h/m*_n_*c*	λ_C,n_	1.31959110(12)	10^−15^ m	0.089
*λ*_C,n_/2*π*	λ_C,n_	2.10019445(19)	10^−16^ m	0.089
neutron magnetic moment *	*μ*_n_	0.96623707(40)	10^−26^ J T^−1^	0.41
in Bohr magnetons	*μ*_n_/*μ*_B_	1.04187563(25)	10^−3^	0 24
in nuclear magnetons	*μ*_n_/*μ*_N_	1.91304275(45)		0.24
neutron–electron				
magnetic moment ratio	*μ*_n_/*μ*_e_	1.04066882(25)	10^−3^	0.24
neutron-proton				
magnetic moment ratio	*μ*_n_/*μ*_p_	0.68497934(16)		0.24
Deuteron				
deuteron mass	*m*_d_	3.3435860(20)	10^−27^ kg	0.59
		2.013553214(24)	u	0.012
in electron volts, m_d_*c*^2^/{*e*}		1875.61339(57)	MeV	0.30
deuteron–electron mass ratio	*m*_d_/*m*_e_	3670.483014(75)		0.020
deuteron–proton mass ratio	*m*_d_/*m*_p_	1.999007496(6)		0.003
deuteron molar mass	*M*(d), *M*_d_	2.013553214(24)	10^−3^ kg/mol	0.012
deuteron magnetic moment*	*μ*_d_	0.43307375(15)	10^−26^ J T^−1^	0.34
in Bohr magnetons,	*μ*_d_/*μ*_B_	0.4669754479(91)	10^−3^	0.019
in nuclear magnetons, deuteron–electron	*μ*_d_*/μ*_N_	0.857438230(24)		0.028
deuteron–electron				
magnetic moment ratio	*μ*_d_/*μ*_e_	0.4664345460(91)	10^−3^	0.019
deuteron–proton				
magnetic moment ratio	*μ*_d_/*μ*_p_	0.3070122035(51)		0.017
PHYSICO-CHEMICAL CONSTANTS				
Avogadro constant	*N*_A_, *L*	6.0221367(36)	10^23^mol^−1^	0.59
atomic mass constant, $\frac{1}{12}m{(}^{12}C)$	*m*_u_	1.6605402(10)	10^−27^ kg	0.59
in electron volts, *m*_u_*c*^2^/{*e*}		931.49432(28)	MeV	0.30
Faraday constant	*F*	96485.309(29)	Cmol^−1^	0.30
molar Planck constant	*N*_A_*h*	3.99031323(36)	10^−10^ J s mol^−1^	0.089
	*N*_A_*hc*	0.11962658(11)	J m mol^−1^	0.089
molar gas constant	*R*	8.314510(70)	J mol^−1^ K^−1^	8.4
Boltzmann constant, *R*/*N*_A_	*k*	1.380658(12)	10^−23^J K^−1^	8.5
in electron volts, *k*/{*e*}		8.617385(73)	10^−5^ eV K^−1^	8.4
in hertz, *k/h*		2.083674(18)	10^10^ Hz K^−1^	8.4
in wavenumbers, *k*/*hc*		69.50387(59)	m^−1^ K^−1^	8.4
molar volume (ideal gas), *RT*/*p*				
*T* = 273.15 K, *p* = 101325 Pa	*V*_m_	22.41410(19)	L/mol	8.4
Loschmidt constant, *N*_α_/*V*_m_	*n*_o_	2.686763(23)	10^25^ m^−3^	8.5
*T*= 273.15 K, *p* = 100 kPa	*V*_m_	22.71108(19)	L/mol	8.4
Sackur-Tetrode constant				
(absolute entropy constant), ** $\frac{5}{2}+\ln\left\{{\left(2\pi m_{u}kT_{1}/h^{2}\right)}^{\frac{3}{2}}kT_{1}/p_{o}\right\}$				
*T*_1_ = 1 K, *p*_o_ = 100 kPa	*S*_o_/*R*	−1.151693(21)		18
*p*_o_ = 101325 Pa		−1.164856(21)		18
Stefan–Boltzmann constant, (*π*^2^/60)*k*^4^/*ħ*^3^*c*^2^	*σ*	5.67051(19)	10^−8^ W m^−2^ K^−4^	34
first radiation constant, 2*πhc*^2^	*c*_1_	3.7417749(22)	10^−16^ W m^2^	0.60
second radiation constant, *hc*/*k*	*c*_2_	0.01438769(12)	m K	8.4
Wien displacement law constant, *b = λ*_max_*T* = *c*_2_/4.96511423 …	*b*	2.897756(24)	10^−3^ m K	8.4

* The scalar magnitude of the neutron moment is listed here. The neutron magnetic dipole is directed oppositely to that of the proton, and corresponds to the dipole associated with a spinning negative charge distribution. The vector sum, *μ_d_=μ*_p_*+μ*_n_, is approximately satisfied.

** The entropy of an ideal monatomic gas of relative atomic weight *A*_r_ is given by $S=S_{o}+\frac{3}{2}\;R\;\ln\;A_{r}-R\;\ln\;(p/p_{o})+\frac{5}{2}\;R\;\ln\;(T/K)$.

**Table 3:** Maintained units and standard values A summary of “maintained” units and “standard” values and their relationship to SI units, based on a least- squares adjustment with 17 degrees of freedom. The digits in parentheses are the one-standard-deviation uncertainty in the last digits of the given value. Since the uncertainties of many of these entries are correlated, the full covariance matrix must be used in evaluating the uncertainties of quantities computed from them.

Quantity	Symbol	Value	Units	Relative Uncertainty (ppm)
electron volt, (*e*/C) J = {*e*} J	eV	1.60217733(49)	10^−19^ J	0.30
(unified) atomic mass unit, $1\;u\;=m_{u}=\frac{1}{12}m{(}^{12}C)$	u	1.6605402(10)	10^−27^ kg	0.59
standard atmosphere	atm	101325	Pa	(exact)
standard acceleration of gravity	*g*_n_	9.80665	ms^−2^	(exact)
‘As–Maintained’ Electrical Units				
BIPM maintained ohm, Ω_69–BI_ ω_βι__85_ ≡ Ω_69–BI_(1 Jan 1985)	Ω_BI85_	1 – 1.563(50)×10^−6^ = 0.999998437(50)	Ω Ω	0.050
Drift rate of Ω_69–BI_	$\frac{d\Omega_{69-\mathrm{BI}}}{dt}$	−0.0566(15)	*μ*Ω/a	—
BIPM maintained volt, V_76–BI_ ≡ 483594 GHz(*h*/2e)	V_76–BI_	1 – 7.59(30)×10^−6^ = 0.99999241(30)	V V	0.30
BIPM maintained ampere, A_BIPM_ = V _76–BI_/Ω_69–BI_	A_BI85_	1 – 6.03(30)×10^−6^ = 0.99999397(30)	A A	0.30
X-Ray Standards				
Cu x-unit : λ(CuK*α*_1_) ≡ 1537.400 xu	xu(CuKα_1_)	1.00207789(70)	10^−13^ m	0.70
Mo x-unit : *λ*(MοK*α*_1_) ≡ 707.831 xu	xu(MoKα_1_)	1.00209938(45)	10^−13^ m	0.45
Å*:*λ*(WK*α*_1_) ≡ 0.209100 Å*	Å*	1.00001481(92)	10^−10^ m	0.92
lattice spacing of Si	*a*	0.54310196(11)	nm	0.21
(in vacuum, 22.5 °C), + $d_{220}=a/\sqrt{8}$	*d*_220_	0.192015540(40)	nm	0.21
molar volume of Si, *M*(Si)/*ρ*(Si) = *N*_A_*a*^3^/8	*V*_m_(Si)	12.0588179(89)	cm^3^/mol	0.74

+ The lattice spacing of single-crystal Si can vary by parts in 10^7^ depending on the preparation process. Measurements at PTB indicate also the possibility of distortions from exact cubic symmetry of the order of 0.2 ppm.

**Table 4:** Energy conversion factors To use this table note that all entries on the same line are equal; the unit at the top of a column applies to all of the values beneath it. **Example:** 1 eV = 806544.10 m^−1^

	J	kg	m^−1^	Hz
1 J =	1	1/{c^2^} 1.11265006×10^−17^	1/{*hc*} 5.0341125(30)×10^24^	1/{*h*} 1.509188 97(90)×10^33^
1 kg =	{*c*^2^} 8.987551787×10^16^	1	{*c*/*h*} 4.5244347(27) ×10^41^	{*c*^2^/*h*} 1.35639140(81)×10^50^
1 m^−1^ =	{*hc*} 1.9864475(12)×10^−25^	{*h/c*} 2.210 2209(13)×10^−42^	1	{*c*} 299792458
1 Hz =	{*h*} 6.626 0755(40)×10^−34^	{*h*/*c*^2^} 7.3725032(44) × 10^−51^	1/{*c*} 3.335640952×10^−9^	1
1 K =	{*k*} 1.380658(12)×10^−23^	{*k/c*^2^} 1.536189(13)×10^−40^	{*k*/*hc*} 69.50387(59)	{*k*/*h*} 2.083674(18)×10^10^
1 eV =	{*e*} 1.60217733(49)×10^−19^	{*e*/*c*^2^} 1.78266270(54)×10^−36^	{*e/hc*} 806554.10(24)	{*e*/*h*} 2.41798836(72)×10^14^
1 u =	{*m*_u_*c*^2^} 1.49241909(88)×10^−10^	{*m*_u_} 1.6605402(10)×10^−27^	{*m*_u_*c/h*} 7.51300563(67)×10^14^	{*m*_u_*c*^2^/*h*} 2.25234242(20)×10^23^
1 hartree =	{2*R_∞_hc*} 4.3597482(26)×10^−18^	{2*R*_∞_*h*/*c*} 4.8508741(29)×10^−35^	{2*R*_∞_} 21947463.067(26)	{2 *R*_∞_*c*} 6.5796838999(78)×10^15^

	K	eV	u	hartree
1 J =	1/{*k*} 7.242924(61)×10^22^	1/{*e*} 6.2415064(19)×10^18^	1/{*m*_u_*c*^2^} 6.7005308(40) ×10^9^	1/{2*R_∞_hc*} 2.2937104(14)×10^17^
1 kg =	{*c*^2^/*k*} 6.509616(55)×10^39^	{*c*^2^/*e*} 5.6095862(17)×10^35^	1/{*m*_u_} 6.0221367(36)×10^26^	{*c*/2*R_∞_h*} 2.0614841(12)×10^34^
1 m^−1^ =	{*hc*/*k*} 0.01438769(12)	{*hc/e*} 1.23984244(37)×10^−6^	{*h/m*_u_*c*} 1.33102522(12)×10^−15^	1/{2*R_∞_*} 4.5563352672(54)×10^−8^
1 Hz =	{*h/k*} 4.799216(41)×10^−11^	{*h/e*} 4.1356692(12)×10^−15^	{*h/m*_u_*c*^2^} 4.43982224(40)×10^−24^	l/{2*R_∞_c*} 1.5198298508(18)×10^−16^
1 K =	1	{*k*/*e*} 8.617385(73)×10^−5^	{*k/m*_u_*c*^2^} 9.251140(78)×10^−14^	{*k/*2*R_∞_hc*} 3.166829(27)×10^−6^
1 eV =	{*e/k*} 11604.45(10)	1	{*e*/*m*_u_*c*^2^} 1.07354385(33)×10^−9^	{*e*/2*R_∞_hc*} 0.036749309(11)
1 u =	{*m*_u_*c*^2^/*k*} 1.0809478(91)×10^13^	{*m*_u_*c*^2^/*e*} 931.49432(28)×10^6^	1	{*m*_u_*c*/2*R_∞_h*} 3.42317725(31)×10^7^
1 hartree =	{2 *R_∞_hc*/*k*} 3.157733(27)×10^5^	{2*R_∞_hc/e*} 27.2113961(81)	{2*R_∞_h/m*_u_*c*} 2.92126269(26)×10^−8^	1

**Table 5:** Expanded covariance and correlation coefficient matrix for the 1986 recommended set of fundamental physical constants The elements of the covariance matrix appear on and above the major diagonal in (parts in 10^9^)^2^; correlation coefficients appear in *italics* below the diagonal. The values are given to as many as six digits only as a matter of consistency. The correlation coefficient between *m_c_* and *N*_A_ appears as −1.000 in this table because the auxiliary constants were considered to be exact in carrying out the least-squares adjustment. When the uncertainties of *m*_p_*/m*_e_ and *M*_p_ are properly taken into account, the correlation coefficient is −0.999 and the variances of *m*_e_ and *N*_A_ are slightly increased.

	α^−1^	*K*_v_	*K*_Ω_	μ_μ_/μ_p_	*e*	*h*	*m*_e_	*N*_A_	*F*
α^−1^	1997	−1062	925	3267	−3059	−4121	−127	127	−2932
*K*_v_	−*0.080*	87988	90	−1737	89050	177038	174914	−174914	−85864
*K*_Ω_	*0.416*	*0.006*	2477	1513	−835	−744	1105	−1105	−1939
μ_μ_/μ_p_	*0.498*	−*0.040*	*0.207*	21523	−5004	−6742	−208	208	−4796
*e*	−*0.226*	*0.989*	−*0.055*	−*0.112*	92109	181159	175042	−175042	−82933
*h*	−*0.154*	*0.997*	−*0.025*	−*0.077*	*0.997*	358197	349956	−349956	−168797
*m*_e_	−*0.005*	*0.997*	*0.038*	−*0.002*	*0.975*	*0.989*	349702	−349702	−174660
*N*_A_	*0.005*	−*0.997*	−*0.038*	*0.002*	−*0.975*	−*0.989*	−*1.000*	349702	174660
*F*	−*0.217*	−*0.956*	−*0.129*	−*0.108*	−*0.902*	−*0.931*	−*0.975*	*0.975*	91727

## Footnotes

1 CODATA was established in 1966 as an interdisciplinary committee of the International Council of Scientific Unions. It seeks to improve the compilation, critical evaluation, storage, and retrieval of data of importance to science and technology. Dr. David R. Lide, chief of the NBS Office of Standard Reference Data, is the current President of CODATA.

2 Figures in brackets indicate literature references.

3 Throughout, all uncertainties are one standard deviation estimates.

4 The variable *d*_220_ is omitted from table 5 because there is little need for its correlations with other quantities. Moreover, since the more significant and related quantity *N*_A_ is included (note that $N_{A}\sim d_{220}^{-3}$), there is no loss of information by omitting *d*_220_.

5 Note that in using eq (3), we set *s* = *k*, $\epsilon_{k}^{2}=v_{kk}$, suppress *k* as a subscript on *Y*, and replace *k* with μ_B._

## References

[1] Cohen ER, Taylor BN (1986). The 1986 Adjustment of the Fundamental Physical Constants, a Report of the CODATA Task Group on Fundamental Constants.

[2] (1973). Recommended Consistent Values of the Fundamental Physical Constants, 1973, a Report of the CODATA Task Group on Fundamental Constants.

[3] Cohen ER, Taylor BN (1973). J Phys Chem Ref Data.

[4] Taylor BN, Parker WH, Langenberg DN (1969). Rev Mod Phys.

[5] Cohen ER, DuMond JWM (1965). Rev Mod Phys.

[6] (1972). Com Intl Poids Mes Com Consult d’Electricité, Trav 13º Session.

[7] Séances PV (1972). Com Intl Poids Mes, 61º Session.

[8] Taylor BN (1986). J Res Natl Bur Stand.

[9] Taylor BN (1987). J Res Natl Bur Stand.

